# High Throughput Human T Cell Receptor Sequencing: A New Window Into Repertoire Establishment and Alloreactivity

**DOI:** 10.3389/fimmu.2021.777756

**Published:** 2021-11-05

**Authors:** Jianing Fu, Mohsen Khosravi-Maharlooei, Megan Sykes

**Affiliations:** ^1^ Columbia Center for Translational Immunology, Department of Medicine, Columbia University, New York, NY, United States; ^2^ Department of Surgery, Columbia University, New York, NY, United States; ^3^ Department of Microbiology & Immunology, Columbia University, New York, NY, United States

**Keywords:** human alloresponse, organ transplantation, high throughput TCR sequencing, human immune system mouse models, thymic selection, immune tolerance, public T cell receptors

## Abstract

Recent advances in high throughput sequencing (HTS) of T cell receptors (TCRs) and in transcriptomic analysis, particularly at the single cell level, have opened the door to a new level of understanding of human immunology and immune-related diseases. In this article, we discuss the use of HTS of TCRs to discern the factors controlling human T cell repertoire development and how this approach can be used in combination with human immune system (HIS) mouse models to understand human repertoire selection in an unprecedented manner. An exceptionally high proportion of human T cells has alloreactive potential, which can best be understood as a consequence of the processes governing thymic selection. High throughput TCR sequencing has allowed assessment of the development, magnitude and nature of the human alloresponse at a new level and has provided a tool for tracking the fate of pre-transplant-defined donor- and host-reactive TCRs following transplantation. New insights into human allograft rejection and tolerance obtained with this method in combination with single cell transcriptional analyses are reviewed here.

## Introduction

The extraordinary magnitude of the T cell alloresponse is the most significant barrier to successful organ and hematopoietic cell transplantation. The ease with which strong alloresponses can be elicited by naïve T cell populations *in vitro* in mixed lymphocyte reactions (MLRs) and cell-mediated lympholysis (CML) assays reflects the unusual strength of this response, in contrast to responses against pathogens, which typically require priming in order to elicit measurable *in vitro* responses. In order to overcome this alloresponse and prevent graft rejection and graft-vs-host disease (GVHD), which occur predominantly in solid organ and hematopoietic cell transplantation, respectively, high levels of immunosuppressive therapy are required. While considerable advances have been made in using immunosuppression to prevent rejection and GVHD in recent years, the consequences of this immunosuppression include susceptibility to opportunistic infections and malignancies. Furthermore, chronic rejection and GVHD persist as common complications of organ and hematopoietic cell transplantation, respectively, despite these advances. All of these difficulties reflect, in large part, the unusual features of alloresponses compared to typical immune responses to pathogens.

In contrast to responses to pathogens, which involve recognition of a limited number of peptides presented by an individual’s own major histocompatibility complex (MHC) molecules, the largest component of the alloresponse is directed against highly polymorphic MHC alleles. Human leukocyte antigens (HLA), the MHC antigens specific to humans, arise from more than 220 genes located on the short arm of human chromosome 6, and include more than 25,000 alleles (1). This polymorphism is believed to have evolved to allow sufficient diversity within the species so that T cells of at least some individuals can respond to new infections without the entire species being eradicated by new organisms that might succeed in evading a less varied immune response.

T cell receptors (TCRs), which recognize peptide antigens bound in the groove of MHC molecules, evolved for this interaction with MHC molecules. Antigen recognition is determined by the TCR complementarity determining regions (CDRs) encoded by V, D and J regions along with nucleotide (N) insertions. In αβ T cells, a TCR α and β chain form a heterodimer. Both chains contain a hypervariable CDR3 region reflecting somatic recombination and N insertions, leading to tremendous TCR diversity (2). Accumulating studies suggest that TCR allorecognition is driven by recognition of the combination of foreign HLA alleles and the peptides they bind (3, 4). It is likely that myriads of allogeneic HLA/peptide molecule specificities are recognized as alloantigens. This assumption is consistent with the very high proportion and number of unique alloreactive TCR sequences that have been detected in TCR sequencing studies (5). An understanding of alloreactivity requires an in-depth understanding of the process of T cell repertoire selection in the thymus. Such studies have been performed almost uniquely in mice, resulting in the view that alloreactivity is due to a combination of factors involved in thymocyte selection: 1) the intrinsic affinity of pre-selection TCR molecules for MHC molecules (6); 2) positive selection, in which only thymocytes with TCRs that exceed a threshold binding affinity to a “self” MHC/peptide complex are rescued from programmed cell death (7); 3) the high level of degeneracy of TCR recognition, such that multiple disparate peptide/MHC complexes can bind a single TCR (8); 4) negative selection in the thymus, which removes only T cells with receptors binding strongly to “self” (not allogeneic) MHC/peptide complexes (9). The net result of this situation is selection of a T cell repertoire with low affinity for self/MHC but no limitation on the affinity for allogeneic MHC/peptide complexes, which are not present during negative selection. While it is likely that these same principles apply to human T cell development and repertoire selection, manipulation of experimental variables is far less achievable for studies in the human than the murine species, resulting in much less detailed information on these processes.

The development in recent years of next generation, high throughput TCR sequencing (10) provides a new opportunity to understand development of the human T cell repertoire, alloreactivity and mechanisms by which autoreactivity is avoided. In this review, we focus on the use of this tool to allow direct analysis of the human alloreactive TCR repertoire and track it in transplant recipients. We not only cover the repertoire analysis of human conventional αβ T cells, but also of unconventional γδ T cells, which tend to have a more tissue-driven clonal distribution, with Vδ2^+^ clonotypes dominating the circulation and Vδ2^-^ clonotypes dominating the non-lymphoid tissues (11). We also summarize studies using this methodology to understand the development of human TCR repertoires in human immune system (HIS) mouse models and how this understanding bears on autoreactivity and alloreactivity.

## Understanding Human T Cell Repertoire Development and Selection Using High Throughput TCR Sequencing in Combination With HIS Mouse Models

### The Process of TCR Repertoire Formation in the Human Thymus

Development of a diverse TCR repertoire in the thymus is needed to provide T cell-mediated protection against a wide range of pathogens. It has been suggested that, as a result of somatic rearrangements and pairing of α and β TCR chains, 10^15^ to 10^18^ unique TCRαβ sequences could theoretically form (12, 13). However, only 2 × 10^11^ T cells exist in a human body (12, 13). With so much potential diversity and limited space for T cells in the peripheral immune system, trimming of the repertoire during positive and negative selection processes in the thymus plays an important role (14, 15). These selection processes ensure optimal self MHC-restricted T cell responses to foreign peptides and prevent the development of autoimmune diseases, respectively. A next generation sequencing study estimated that there are approximately 100 million unique TCRβ sequences in a young adult human, which declined two- to five-fold in healthy elderly people. This age-related contraction was less pronounced for memory than naïve T cells (16), reflecting the decline in thymic function with age. Another deep sequencing study has estimated that there are 40 × 10^6^ to 70 × 10^6^ unique TCRβ sequences and 60 × 10^6^ to 100 × 10^6^ TCRα sequences in human pediatric thymi (17).

Diversity of the TCR repertoire is formed at different levels, including random recombination of V, (D) and J genes for both TCRα and β chains, addition and deletion of random nucleotides at V (D) J junctional sites mediated by the enzyme terminal deoxynucleotidyl transferase (TdT) in the β chain, and pairing of TCRα and β chains (18). The processes of T cell selection in the thymus are still not completely understood, with vastly more information being available from murine studies than from humans.

**Figure 1:**
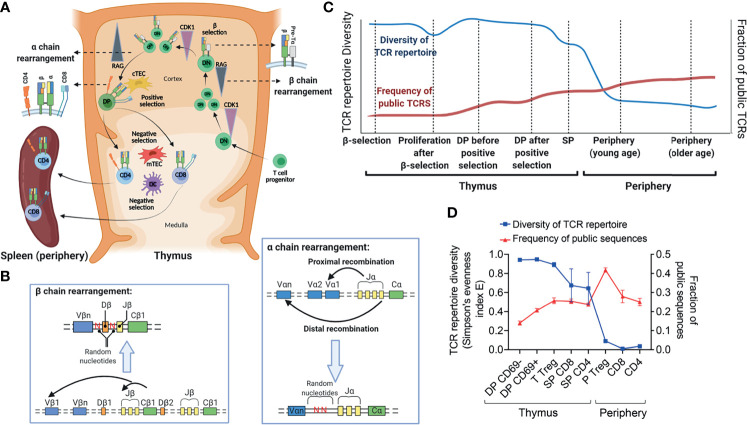
TCR repertoire formation in human thymus and periphery. **(A)** Schematic presentation of different stages of T cell development in human thymus and periphery. **(B)** TCR β and α chain rearrangement processes. **(C)** Hypothetical changes in “TCR repertoire diversity” and “frequency of public TCRs” through different developmental stages. **(D)** Kinetics of changes in the diversity of TCRβ repertoire, as measured by the Simpson’s evenness index E, and fraction of public sequences in different subsets of human T cells from the thymus and periphery of HIS mice generated by transplantation of human thymocyte-depleted fetal thymus tissue and fetal liver HSCs into immunodeficient NSG mice (n=2-7). Public sequences were defined as TCRβ CDR3 sequences that were repeated between different mice or different cell subsets (19). This figure was created with BioRender.com.

Early in the T cell development process in the thymus, recombination activating enzymes RAG-1 and RAG-2 are upregulated and V, D and J genes of a β chain gene recombine, permitting β selection to occur (**Figure 1A**). In human fetal thymocytes, RAG enzymes were detected after a round of replication during the double negative (DN) and another round of replication during the double positive (DP) stage (20). Upon productive rearrangement, the formed β chain pairs with a pre-TCRα molecule and relays a signal for proliferation to the developing T cell at the late DN proliferative stage (20, 21) (**Figure 1A**). After several rounds of proliferation, RAG enzymes are expressed again and V and J genes of an α chain recombine, allowing CD4^+^ CD8^+^ DP cells to be formed. The expression of an αβ TCR allows these DP cells to undergo positive and negative selection and differentiate into single positive (SP) CD4 and CD8 T cells (**Figure 1A**). A fully formed polyclonal β chain repertoire is detected at the DP CD3^-^ stage, while polyclonal α chain rearrangements are only evident at the DP CD3^low^ stage and later (22). It is estimated that only 3% of thymocytes reach a mature state and exit the thymus (22).

For human thymocytes, the ratio of nonproductive to productive recombination events was higher prior to the proliferative phase of the DN stage, indicating that thymocytes that fail to generate a productive β chain for the first allele try another recombination with the other allele. However, the ratio of nonproductive recombinations was not increased for TCRα during the DP stage, suggesting that cells with a nonproductive TCRα may not undergo a second allele recombination, but instead die (20). However, murine studies have demonstrated that a second productive α chain rearrangement can occur (23, 24), resulting in the presence of two α chains and two alternative TCRs in one thymocyte. Observations in human T cells (25) and thymocyte selection studies (26) indicate that a similar situation pertains.

Studies of peripheral human TCR repertoires have established that CD4 cells are more diverse compared to CD8 T cells (16, 27–29), possibly due to differences between the subsets in thymic output and/or levels of clonal expansion/persistence in response to infections.

### Genetic Influences on TCR Repertoire Selection

By comparing TCRβ repertoires between different individuals, the level of sharing of TCRβ sequences was found to be considerably higher than that expected based on the number of clonotypes (30). The level of TCRα sharing has been shown to be even larger than TCRβ sharing among healthy humans (31). Therefore, there may be genetic factors or selection forces that drive the sharing of certain TCR sequences. However, one limitation of most of these studies is their reliance on analysis of peripheral T cell repertoires rather than thymocytes, which are less accessible. Peripheral TCR repertoires have undergone post-thymic selection that reflects environmental factors such as antigenic exposures, potentially obscuring the role of genetic factors.

Monozygotic twin studies have been used in efforts to gain insight into the role of genetic factors in TCR repertoire generation in humans. All of these studies point to a very diverse and divergent TCR repertoire with low levels of sharing between unrelated individuals and even between identical twins. One analysis of peripheral TCR repertoires of three pairs of monozygotic twins revealed similar degrees of sharing between identical twins and unrelated individuals (32). However, CDR3 sharing between identical twins was significantly higher within the fraction of highly abundant TCRs. Analysis of out-of-frame sequences provides a window into the TCR repertoire prior to selection, as these sequences are not subjected to selection processes. In this study, V gene usage in out-of-frame sequences was more similar in identical twins than unrelated individuals, pointing to a genetically determined bias prior to selection (32). Another study involved high throughput single T cell sequencing on 15 healthy individuals, including 6 monozygotic twin pairs. Although sharing of entire TCR including α and β chains was infrequent, such sharing was greater between identical twins than between others, which could suggest that genetic components have a role in TCR repertoire selection or could point to shared environmental exposures (33).

There is controversy in the literature about the relative roles of genetic background and thymic selection in determining V (D) J gene usage (32, 34–37). Overall, a general pattern is shared throughout human populations, such that certain genes are always used more than others (38). However, genetic factors lead to subtle differences. To discover the associations between genetic variation and TCR V gene usage, Sharon et al. applied expression quantitative trait locus (eQTL) mapping in a large human cohort and discovered associations between variation in the HLA locus and TCR V gene usage (39). Other studies established increased similarity in V and J gene usage between identical twins in both peripheral (40) and thymic T cells (41), despite the overall similarity between any two individuals. A more recent study using single cell RNA sequencing elucidated further details about the process of V (D) J gene recombination and TCR α and β chain usage (20). For TCR β, an overall pattern of VDJ recombination that was established during the DN stage (after β selection) was shown to persist during subsequent developmental stages in the thymus. This pattern was correlated with the genomic positions and looping structures of different loci. Although this initial pattern formed the overall repertoire, thymic selection led to enrichment or deletion of some Vβ genes at developmental stages following β selection. On the other hand, an association between developmental timing and V-J pairing was detected for the TCRα locus. Proximal V-J pairs were formed in early stages, followed by distal pairs in later stages. Proximal pairs declined relatively as thymic selection progressed, which points to progressive recombination of the TCRα locus (**Figure 1B**). Interestingly, SP CD8 T cells were enriched for the distal pairs, suggesting a slower or less efficient commitment toward the CD8 T lineage (20).

### Role of Recombinatorial Bias vs Thymic Selection in Abundance of Public TCRs

TCRs shared between different individuals are also called “public TCRs”. Sharing of TCRβ chains in peripheral blood CD8 T cells has been shown to be 500 times greater than what is expected from TCR formation from random recombinations (30). High throughput TCRβ sequencing of 80 million TCR sequences from 666 people revealed that around 14% of TCRβs are public (42). Among DP thymocytes in syngeneic mice, about 15% of TCRs were found to be public, and most of them were detected in the preselection repertoire, suggesting recombinatorial bias (43). In another mouse study, CDR3β sequencing performed in TCRα^-/-^ mice, in which functional TCRαβ T cells do not form and therefore thymic selection does not occur, revealed that 11% of sequences were shared (44), again suggesting that recombinatorial bias is largely responsible for development of public TCRs. A third study involved high throughput TCRβ sequencing on a large group of mice, including syngeneic and allogeneic MHCs. Shared CDR3s were enriched among the most abundant TCRs. A group of 289 public CDR3βs constituted about 10% of the whole repertoire. Public CDR3βs were highly convergent (having high ratios of nucleotide sequences per amino acid sequence), utilized restricted V/J segments, were found in mice with different MHCs, and were shorter compared to private sequences. Computer simulations of the VDJ recombination process generated public TCRs similar to the actual dataset. These authors concluded that both recombinatorial bias and thymic selection play a role in the existence of public TCRs (45). A recent study of TCR α and β sequences in a cohort of humans showed that shared sequences are more common in productive compared to nonproductive sequences, pointing to the effect of thymic selection in enrichment of public TCRs, while gene segment usage between unrelated individuals suggested a role for recombinatorial bias as well (38).

Productive human CDR3βs have been shown to be shorter on average than out-of-frame sequences, indicating that thymic selection favors shorter sequences (27). Consistently, direct analyses of murine thymocytes have shown that mouse CDR3αs shorten as thymic selection progresses (46). The observation that public CDR3βs are shorter compared to private ones in human TCRβ repertoires suggests a possible role for thymic selection of public sequences. The observation that the same public sequences develop independently of MHC genotypes both in mice (45) and in humans (32) suggests that these sequences may be crossreactive and can interact with different peptide-MHC (p.MHC) complexes. Additionally, significant CDR3β sharing has been demonstrated between different peripheral blood human T cell subsets (47), supporting the hypothesis that shared sequences are highly crossreactive. A hypothetical increasing pattern of public TCRs during thymic and peripheral selection processes is shown in **Figure 1C**. Our HIS mouse studies, described in *Lessons Learned From HIS Mouse Models* and **Figure 1D**, provide experimental evidence in support of this hypothetical pattern.

### Selection of Crossreactive and Autoreactive TCRs

Accumulating evidence indicates that public TCRs are enriched for autoreactive and cross-reactive sequences and vice versa. In a single cell TCR sequencing study, analysis of more than 3000 gluten-specific CD4 TCR clonotypes isolated from 63 celiac patients showed that gluten-specific autoreactive T cells were enriched for public TCRs (48). By comparing thymic TCRα sequences from 6 human donors to a list of sequences known for their reactivity for type 1 diabetes (T1D)-associated self-antigens and HIV antigens, Heikila et al. showed that autoreactive (T1D-associated) TCRs were generated in significantly higher numbers compared to nonself-reactive (HIV-associated) TCRs in human thymi and were not negatively selected or converted to Tregs before entering the periphery. These autoreactive TCRs were selected by different thymi, independent of their HLA type (49). In a murine study, TCRβ sequencing of T cells in thymus, periphery and central nervous system of mice with experimental autoimmune encephalomyelitis implicated public TCRs in autoimmunity. Interestingly, these autoreactive public TCRs were preferentially identified in the preselection repertoire, pointing to recombinatorial bias as the main factor in their development (50). T cells in T1D patients were shown to have shorter CDR3βs with increased deletions/reduced insertions during VDJ rearrangement compared to healthy controls, even in nonproductive sequences, implicating recombinatorial bias in the development of autoreactive T cells in T1D. CDR3s in autoreactive CD4 T cell clones in these patients were shorter than those in T cells recognizing viral antigens (51).

Flexible CDR3 loops allow TCRs to bind multiple p.MHC complexes and hence be degenerate in their antigen recognition (13, 52). This phenomenon is needed for T cells to recognize a repertoire of p.MHCs that is larger than the repertoire of possible TCRs (13). A single T1D-reactive TCR (1E6) was shown by experimental and computational analysis to be capable of binding to more than a million peptides presented on HLA-A2 (53). Available evidence indicates that shorter TCRs have a greater chance of being promiscuous and cross-reactive. Recombinatorial bias and thymic selection appear to favor these sequences, which are enriched for autoreactive TCRs. Although TCR crossreactivity allows recognition of some pathogens whose peptides are not presented on cortical thymic epithelial cells (cTECs), peptides containing TCR contact amino acid motifs not present in the human proteome were recently reported to be unrecognizable by naïve human T cells, resulting in potential holes in the TCR repertoire for recognition of potential pathogens (54).

TCR repertoires of CD4 and CD8 T cells have been found to have little CDR3β overlap and to have distinct characteristics, including the preference for certain amino acids in their CDR3βs (29). However, a recent single cell TCR sequencing study detected overlap between TCR repertoires of CD4 and CD8 T cells of the same individuals at CDR3α (7.8%), CDR3β (4.7%) and even in paired αβ (0.65%) (55). Although the overlapping repertoire at the paired αβ level is minimal, it might be enriched for cross-reactive sequences that are capable of recognizing both MHC-I and II molecules, perhaps with sufficient affinity that coreceptor expression is irrelevant. In the section on HIS mouse models below, we discuss evidence that public CDR3β sequences may be associated with such activity, as the same amino acid sequences are frequently detected among both CD4 and CD8 populations and in the context of completely disparate thymic HLA molecules (19).

### Lessons Learned From HIS Mouse Models

Access to human thymus tissue is limited and, in contrast to mice, genetics and other variables cannot be readily manipulated in order to study the development of the human T cell repertoire. HIS mouse models involving transplantation with human fetal thymus tissue along with human hematopoietic stem cells (HSCs), however, provide an opportunity to study the factors involved in human TCR repertoire selection. Several studies have used HIS mouse models to study V(D)J recombination and CDR3 length distribution (56–59). These studies established that the human TCR repertoire in these HIS mice is diverse and has normal CDR3 length distributions.

In order to investigate the factors involved in human TCR repertoire formation, we recently performed high throughput TCRβ sequencing and single cell TCR sequencing on thymic and peripheral T cells of HIS mice generated with human fetal liver HSCs combined with either autologous or HLA-mismatched allogeneic human fetal thymus tissue (19). Measures were taken to deplete the grafted fetal thymus tissue of pre-existing thymocytes so that synchronized thymic development from progenitors could be investigated in the mice. To ensure that no human thymopoiesis occurred in the native mouse thymus of recipient NSG mice, which could confound interpretation of peripheral TCR repertoire data, we used a rapid method to thymectomize these mice (60).

Our studies revealed that the TCRβ repertoire of different human thymocyte populations is highly diverse (19) (**Figure 1D**). Formation of the human TCR repertoire was shown to be largely stochastic and was almost totally divergent between animals reconstituted simultaneously with identical HSCs, thymus, genetic backgrounds and environment. This finding may help to explain the incomplete penetrance of genetically-controlled autoimmune diseases in identical twins (61). In contrast to the original fetal thymic tissue, in which expression of the TdT enzyme was low, this enzyme was well-expressed in grafted human thymi after they matured, especially in DP thymocytes. Consequently, diversity of the TCR repertoire was significantly greater in grafted human thymi compared to the original human fetal thymus (19). Diversity was lower at the amino acid sequence level compared to the nucleotide sequence level and also in productive sequences compared to non-productive sequences for populations that had undergone selection, demonstrating that thymic selection narrows the TCR repertoire, both at the DP and the SP stages of development. Diversity of TCR repertoire decreased as thymic selection progressed and also further decreased in peripheral compared to thymic SP T cell populations (19) (**Figure 1D**).

We also examined the development of public TCRβ CDR3 sequences using the HIS mouse model. An increase in the proportion of these sequences was observed in the transition from unselected (CD69-) to positively selected (CD69+) DP thymocytes, indicating a bias for their positive selection. We also observed a trend toward an increase in the proportion of shared sequences in SP compared to DP thymocytes and in the peripheral compared to thymocyte SP repertoires (19) (**Figure 1D**). This trend suggests that both positive and negative selection processes, as well as peripheral selection, favor public sequences. The proportion of overlapping CDR3βs was not different between or among autologous (to the HSCs) and allogeneic thymi and many CDR3βs were shared between CD4 and CD8 SP thymocyte subsets, suggesting that the shared CDR3βs, like those discussed above, are cross-reactive. The shared sequences had shorter CDR3β lengths, and we observed that the length of more abundant CDR3βs decreased during thymic selection, indicating that HLA-peptide-driven selection of human thymocytes favors shorter sequences, likely due to increasing cross-reactivity of shorter CDR3s. By comparing the shared and unshared CDR3βs with a repertoire of known T1D autoreactive CDR3β sequences, we found direct evidence that the shared sequences are enriched for autoreactive sequences. Furthermore, they were greatly enriched for CDR3βs that were found in a small subset of TCRs that recognized two completely HLA-mismatched allogeneic stimulator populations, indicating that they are crossreactive to different alloantigens (19). We were able to specifically analyze the impact of selection on shared human thymocyte sequences and demonstrated that thymic selection is a significant factor driving their abundance among mature T cells.

It has been shown in a murine model that TCRβ CDR3 hydrophobicity at position 6 and position 7 (P6 and P7), the residues that interface with the p.MHC, correlated with the ability to be activated by self p.MHC (62). In our HIS mouse studies, we observed that as selection progressed, hydrophobic amino acids were enriched at position 6 and 7 of CDR3β, suggesting a role for self-peptides in human thymocyte selection. However, relatively lower hydrophobicity at P6/P7 in the shared sequences suggested that they may have weaker interactions with self-peptides than unshared sequences and that this may allow them to escape negative selection (19).

Despite the very divergent CDR3β TCR repertoires, we found that V and J gene usage in different cell populations in human thymi was overall very similar among different mice generated with different HSCs and different thymi. However, our study was not powered to detect subtle differences in V gene usage such as those that have been observed in association with HLA alleles. Our analysis of thymocyte subsets demonstrates a significant but minor role for thymic selection in determining ultimate V gene usage. Additionally, we found that the observed VJ pairing pattern correlated very well with what would be expected from stochastic VJ pairing according to their frequencies, suggesting that no major factor biases the process of TCRβ V-J recombinations (19).

### Additional Platforms for Understanding TCR Selection

High throughput methods for paired sequencing of TCR α and β chains will be needed to study selection of clonotypes in high quantities. Existing approaches for simultaneous sequencing of both TCR α and β chains are expensive and have relatively low throughput (20, 63). Development of computational methods to cluster TCR sequences according to their likely p.MHC specificity could be ground-breaking in understanding thymic TCR selection processes. GLIPH (grouping of lymphocyte interactions by paratope hotspots) was one of the first algorithms that was developed for this purpose and has the capability to group raw TCR sequences into likely p.MHC specificity groups (64). While the degeneracy of TCR recognition limits the ability to identify specific peptide antigens with this approach, the clustering may provide a fingerprint of the forces driving thymocyte selection and alloreactivity. Further progress in this area (65) is likely to greatly enhance our understanding of this process.

## Assessment of Human Alloresponses After Transplantation Using High Throughput TCR Sequencing

### The Challenge of Measuring and Tracking Human Alloresponses

Alloresponses, immune responses with unique strength and diversity, are directed to non-self antigens from members of the same species due to extensive genetic polymorphisms between allogeneic donors and recipients. Of primary importance in driving the strength of the alloresponse is the MHC (66), as discussed earlier in this article. Consequences of allorecognition in transplantation include graft rejection and GVHD, two major barriers to the greater success of solid organ and hematopoietic stem cell transplantation.

Studies to track alloresponses *in vivo* in rodents utilized tools like endogenous superantigen-specific Vβs and alloreactive transgenic TCRs. Intrathymic deletion was thereby demonstrated as a major mechanisms of tolerance in mixed or fully allogeneic chimeric mice (67–69). Given the inability to perform such studies *in vivo* in humans, efforts have been made to study a small population of donor-reactive T cells *via* tetramers with known HLA-peptide specificities (70, 71) or to use *in vitro* functional assays, such as MLRs (72, 73), CML assays (74), limiting diluting assays (LDAs) (75) and enzyme-linked immunospot (ELISPOT) assays (76, 77), under particular culture conditions. However, the enormous number of putative HLA/peptide combinations potentially recognized by alloreactive TCRs precludes using the tetramer approach to study a significant fraction of the alloreactive repertoire. *In vitro* functional assays, on the other hand, cannot replicate the *in vivo* setting and have demonstrated inconsistent correlations with clinical outcomes (74, 78–80). Previous attempts to analyze repertoire diversity using conventional methods, including PCR-based CDR3 length analysis by spectratyping and flow cytometry-based V region usage, were limited by low resolution (81–83). Recently, the availability of next-generation sequencing (NGS) technologies (84–86) has opened up new possibilities for understanding this immune repertoire and to tackle fundamental questions of T cell alloimmunity in humans.

### Establishment of an Alloreactive T Cell Clonal Tracking Platform and Application in Normal Donors and Patients Receiving Solid Organ Transplantation (SOT)

The availability of high-throughput sequencing platforms for TCRβ CDR3 sequencing allowed our group to develop a novel approach to specifically identify alloreactive T cell repertoires before transplantation (Tx). Combing CFSE-MLR and high-throughput TCRβ CDR3 DNA sequencing (TCRβ-seq) allows us to track alloreactive TCR clones in different tissues following the Tx without relying on any post-Tx functional readout (80). In normal healthy donors, we measured the clonal frequencies, specificity and repertoire diversity of alloreactive CD4 and CD8 T cells. Our data showed that the alloimmune repertoire is highly specific for a given pair of individuals, with only a few exceptional TCRβ CDR3s crossreacting on disparate HLAs (5). Most alloreactive clones are present at low frequency in the circulation, reflecting the naïve phenotype of many alloreactive T cells. The diversity of this alloreactive repertoire is quite high and increases with increased HLA mismatching. Our analysis of the contribution of naïve and memory T cells to the alloresponse (5) confirmed previous functional studies in which it was concluded that both types of T cells contribute to alloreactivity measured *in vitro* (87, 88). Although the diversity of memory T cell repertoires is much lower than that of naïve T cells (89), pathogen-specific memory T cells can cross-react with alloantigens (90).

We developed a statistical model to estimate the total frequency of both detected and undetected alloreactive clones within the unstimulated circulating repertoire. With this approach, we estimated that a substantial fraction of human circulating TCR repertoire (0.5%-6%) has alloreactive potential to respond to just two different allogeneic stimulators and thereby inferred that most human TCRs likely have potential alloreactivity against some allogeneic HLA alleles (5).

This CFSE-MLR + TCRβ-seq approach (91) has been utilized to investigate human alloresponses and validated in recipients of several different types of organ transplantation, including combined kidney and bone marrow transplantation (CKBMT) (80), conventional kidney transplantation (92), liver transplantation (93), and intestinal transplantation (ITx) (94–96). Through these studies, we have validated the ability of our assay to identify biologically relevant alloreactive T cell clones in pre-Tx MLR that are involved in rejection and expand in patients following transplantation.

In tolerant patients in an Immune Tolerance Network (ITN)-sponsored CKBMT study (ITN036ST), we observed significant and specific long-term declines in the number of circulating donor-reactive T cell sequences. This decline, in contrast to functional assays, distinguished the tolerant patients from a patient on the same protocol who failed to achieve tolerance (80). These studies clearly distinguished clonal deletion from anergy, a distinction which often cannot be on the basis of *in vitro* functional assays. Expansion of circulating donor-reactive sequences was detected in conventional kidney Tx recipients and did not appear to correlate with rejection (80, 97).

We tracked donor‐reactive TCRβ sequences from a group of tolerant (n=3) and nontolerant (n=5) patients from a randomized liver transplantation clinical trial ITN030ST and obtained evidence consistent with deletion of donor‐reactive T cells post-Tx in both groups (93). The fact that clonal reductions were greater in donor-reactive TCRβ sequences than third party-reactive sequences in all 8 subjects, regardless of successful early immunosuppression withdrawal, demonstrates an impact of the liver allograft on the donor-reactive T cell repertoire and suggests that non-deletional mechanisms may be decisive in determining outcomes of immunosuppression withdrawal following liver Tx. The difference between this result and the observation of deletion only in tolerant patients in the CKBMT study highlights the protocol- and organ-specific nature of tolerance mechanisms in humans and the need to avoid considering allograft tolerance as a single entity.

In patients receiving ITx, we found that a faster rate of donor T cell replacement by recipient T cells in the graft mucosa correlated with early rejection, which was associated with a preponderance of host-versus-graft (HvG) T cell clones within the graft, demonstrating the biological significance of the TCRs identified as donor-reactive by pre-transplant MLR (94). Given the high lymphoid cell load carried within intestinal allografts, we hypothesized that clinical outcomes in ITx are largely determined by the exchange of donor and recipient lymphoid tissue and hence the balance of graft-versus-host (GvH) and HvG-reactive T cells. Using CFSE-MLR and the high-throughput TCRβ-seq-based approach to track alloreactive T cells in the GvH and HvG directions in the graft, circulation and bone marrow (BM), we obtained data consistent with this hypothesis (96). ITx provides a unique opportunity to study two-way alloresponses in humans and we did so by applying immunogenomic tools, including single cell sequencing techniques, to enhance our understanding of immune mechanisms after ITx, as detailed in the following sections.

### Immunogenomics Insights Into Bidirectional Alloresponses in Human ITx

ITx serves as the only long-term option for patients who suffer intestinal failure and have functional disorders (98, 99). However, the success of ITx is currently limited by high rejection rates, by the risk of GVHD and by infections and posttransplant lymphoproliferative disease (PTLD) resulting from high levels of immunosuppression (100, 101). Previous reports suggest that composite allograft transplants, such as multivisceral transplantation (MVTx) and liver-intestinal transplantation (LITx), are associated with reduced rates of intestinal rejection compared to isolated intestinal transplantation (iITx) (102, 103). Our own data have confirmed this observation in association with high levels of multilineage peripheral blood chimerism in MVTx recipients (94–96, 104).

Remarkably, we detected the presence of multilineage peripheral blood chimerism in many of these recipients and, further, demonstrated that this chimerism reflects *de novo *lymphopoiesis from hematopoietic stem and progenitor cells (HSPCs) carried in the intestinal allograft (95). We showed that high levels of peripheral blood T cell mixed chimerism (macrochimerism: ≥4% peak donor T cells in recipient PBMCs) occurs commonly, without GVHD, in recipients of intestinal allografts, and is associated with significantly reduced graft rejection, less *de novo *donor-specific antibody (DSA) development and slower recipient T cell repopulation in the graft (94–96, 104). The association between blood T cell macrochimerism and slower graft T cell replacement suggests a connection between immunologic events in the graft (locally) and the blood (systemically).

We investigated the underlying mechanisms of these phenomena at both the cellular and the clonotypic levels, using our published approach of combining CFSE-MLR and high-throughput TCRβ-seq  (80) to track alloreactive T cells in the GvH and HvG directions in the graft, circulation and BM (96). These studies have provided new insights into the significance of donor T cell macrochimerism in blood, including the first direct demonstration of lymphohematopoietic graft-versus-host responses (LGVHR) in humans, in which the GvH response confined to the lymphohematopoietic system promotes engraftment without causing GVHD, a disease that requires T cell migration into epithelial tissues. We found that patients with macrochimerism have high GvH to HvG T cell clonal ratios in their allografts. These GvH clones enter the circulation, where their peak levels are associated with declines in HvG clones early post-Tx, suggesting that LGVHR may contribute to chimerism and control HvG responses systemically. Consistent with the notion that LGVHR creates hematopoietic “space” in the BM for donor hematopoietic cells, as described in murine studies (105, 106), donor-derived T cells, including GvH clones and CD34^+^ HSPCs, were simultaneously detected in the recipients’ BM >100 days post-Tx (96). Individual GvH clones were detected in either the ileal mucosa or PBMCs before detection in recipient BM, consistent with an origin in the intestinal allograft, where donor GvH-reactive T cells expand early upon entry of recipient antigen-presenting cells (APCs) into the graft. These results, combined with cytotoxic single-cell transcriptional profiles of donor T cells in recipient BM, suggest that tissue-resident GvH-reactive donor T cells develop effector function and migrate into the recipient circulation and BM, where they promote engraftment of graft-derived HSPCs and maintain mixed chimerism.

### Integrated TCRαβ-Seq, scRNAseq and Pre- and Post-Tx MLRs to Study Tolerance and Rejection Mechanisms in Human ITx

Outcomes of ITx are suboptimal in large part due to high rejection rates. We have previously demonstrated that pre-Tx MLR-defined HvG alloreactive T-cell clones are enriched in intestinal allografts during early rejection and persist long-term post-Tx despite rejection resolution (94). Recipient T cells in the mucosa eventually acquire tissue resident memory (TRM) features and seed the entire gastrointestinal tract, likely posing a constant threat of late rejection (94). CD8 intraepithelial lymphocytes (IELs) show a TRM phenotype during quiescence (CD69^+^CD103^+/-^CD28^low^), but regain high CD28 expression, despite maintenance of the CD103 and CD69 TRM markers, during late rejection, indicating a possible transitional phenotype of TRM and effector T (Teff) cells that correlates with rejection. Therefore, we hypothesize that HvG-reactive T cells join the TRM pool and may become Teff cells and/or be tolerized. Alternatively, HvG clones developing *de novo* after ITx may contribute to late rejection.

We have established a comprehensive platform to integrate T cell clonal tracking, alloreactivity and RNA profiling to allow us to directly address these hypotheses. In ongoing studies, we have carried out single cell analysis of recipient T cells in the graft mucosa (107). Single cell RNA sequencing (scRNA-seq) was performed using the 10x Genomics platform and the Seurat analysis pipeline (108) for simultaneous measurement of mRNA expression and paired V(D)J TCR α and β sequences at the single cell level. Our published protocol using Adaptive Biotechnologies TCRβ bulk sequencing to identify HvG-reactive and nonHvG-reactive TCR repertoires (80, 94) from pre-Tx recipient lymphoid tissues was applied and single cell TCRβ sequences from intestinal allograft mucosal specimens were mapped to these pre-Tx sequence sets. This methodology allows annotation of each cell, identifying CD4, CD8, HvG and nonHvG clones of each type. To further extend our ability to investigate the induction of immune tolerance, we also set up post-Tx MLR assays using recipient PBMCs post-Tx as responders against donor pre-Tx stimulators and sorted the CFSE^low^ CD4 and CD8 recipient T cells, allowing us to define clones by post-Tx MLR. Therefore, we can separately track the gene expression profiles of the pre-Tx and post-Txdefined HvG clones and assess their contribution to graft rejection or tolerance at the single cell level. We can further investigate the potential genes, pathways and networks involved with functional categories of HvG clones that corelate with immune tolerance and graft rejection after human ITx (107). This integrated platform could be applied to other types of SOT as well as hematopoietic cell transplantation to understand human alloresponses at a deeper level.

### Combined TCRγδ-Seq and scRNA-Seq Approach to Study Unconventional γδ T Cells in Human SOT

γδ T cells can recognize diverse antigens and exert disparate functions that include immune surveillance, modulation, and direct cytotoxicity (109). The innate- and adaptive-like features of human γδ T cells may be driven by differential γδ TCR repertoires, generally defined as innate-like Vγ9^+^δ2^+^ and “adaptive” non-Vγ9δ2, respectively (11). The repertoire can be shaped by tissue compartmentalization, age and history of antigen exposure (11, 110–112). Despite comprising a significant proportion of resident T cells in many organs, including gut and liver, γδ T cells and their possible role in transplantation outcomes are largely under‐researched (113).

Earlier functional studies were limited to *in vitro* systems of human peripheral blood-derived γδ T cells (114, 115), which are dominated by Vδ2 clonotypes. γδ T cells had been shown to exhibit either direct veto-type suppression of alloreactions (114), or indirect stimulation of alloreactive αβ T cell proliferation by inducing maturation of autologous dendritic cells and B cells into functional APCs (115). Tissue-resident Vδ1 T cells in liver explants have been investigated among patients who underwent liver transplantation, and were found to be polyfunctional and found to respond to both TCR and cytokine stimuli *in vitro* (116).

Our ongoing studies (117) suggest that γδ T cells may not only participate in host immune defense, but also likely have the potential to modulate two-way alloreactivities in the graft and hematopoietic system after human ITx. Our regimen of serial biopsies for ITx recipients allows us not only to investigate the role of intragraft γδ T cells in rejection but also to track the dynamics of their homeostatic turnover in the absence of rejection, providing a unique opportunity to study the fundamental biology of human γδ T cells. We have established a novel pipeline to integrate iRepertoire (γδ T cell primer sets) and 10x Genomics (5’cDNA library) platforms to relate functional gene profiles within individual γδ T cell clonotypes. TCR-seq of pre- and post-Tx γδ T cells in intestinal grafts, peripheral blood and BM are performed at the bulk and/or single cell levels. Clonal tracking of γδ T cells in intestinal grafts, peripheral blood and BM at both early and late time points post-Tx will provide a deeper understanding of their tissue origin, migration pattern and phenotypic maturation.

Our platform provides an opportunity to obtain a deeper understanding of the mechanisms behind γδ T cell chimerism, maturation of TRM features, and their modulatory roles on local and systemic alloresponses, facilitating the development of novel strategies to regulate γδ T cells to overcome rejection, infection and increase the utilization of ITx as a life-saving, quality of life-improving procedure. This approach can also be extended to other types of SOT and hematopoietic cell transplantation to provide immunogenomics insights into γδ T cells and their role in modulating alloresponses.

## Conclusions

Advances in sequencing technologies have brought our ability to understand human TCR repertoire establishment and alloreactivity into a new era. Combining these techniques with HIS mouse models allows, for the first time, manipulation of variables that control human T cell development and selection in the human thymus that has and will continue to greatly enhance our understanding of these processes. Integration of immune repertoire profiling and bioinformatic-based prediction of antigen recognition by TCRs with clinical diagnoses of rejection, infection and malignancies will greatly expand our understanding of the mechanisms of major complications after transplantation and promote the development of effective tailored preventive methods and therapies. Additionally, the post-Tx functional genomic analysis of pre-Tx-defined alloreactive TCRs will not only enhance our knowledge of rejection mechanisms but will also point the way toward novel approaches to tolerance induction. Alloreactive TCR identification and tracking also has great potential to enhance our understanding of GvH-reactive T cell compartmentalization in the context of hematopoietic cell transplantation, thereby enhancing our knowledge of GVHD pathogenesis and suggesting novel therapeutic and prophylactic approaches.

## Author Contributions

JF, MK-M, and MS collected data and wrote the review. All authors contributed to the article and approved the submitted version.

## Funding

Studies related to human immune system mouse models were supported by National Institutes of Health (NIH) grants: National Institute of Allergy and Infectious Diseases (NIAID) P01 AI04589716, National Institute of Diabetes and Diseases of the Kidney (NIDDK) R01DK103585, and NIDDK-supported Human Islet Research Network (HIRN) UC4 DK104207 and U01 DK123559 (to MS). Studies related to human organ transplantation were supported in part by NIAID grant P01 AI106697, UM1 AI109565 and National Center for Advancing Translational Sciences (NCATS) grant R21 TR002279 (to MS) and by an American Society of Transplantation Research Network Directed Research Grant #gAST182D0MS (to MS). JF was supported by a Congressionally Directed Medical Research Program (CDMRP) Discovery Award W81XWH-20-1-0159 funded by the Department of Defense (DoD), a R21 grant AI166069 supported by NIH/NIAID and a Nelson Faculty Development Award UR011630-01 from the Nelson Family Transplantation Innovation Award Program at Columbia University Irving Medical Center. MK-M was supported by an American Diabetes Association (ADA) Postdoctoral Fellowship #1-18-PDF-074, a Columbia University Naomi Berrie Diabetes Center Russell Berrie Foundation Fellowship, and a Nelson Faculty Development Award UR011632-01.

## Conflict of Interest

The authors declare that the research was conducted in the absence of any commercial or financial relationships that could be construed as a potential conflict of interest.

## Publisher’s Note

All claims expressed in this article are solely those of the authors and do not necessarily represent those of their affiliated organizations, or those of the publisher, the editors and the reviewers. Any product that may be evaluated in this article, or claim that may be made by its manufacturer, is not guaranteed or endorsed by the publisher.

## References

[1] Robinson J Halliwell JA Hayhurst JD Flicek P Parham P Marsh SG. The IPD and IMGT/HLA Database: Allele Variant Databases. Nucleic Acids Res (2015) 43(Database issue):D423–31. doi: 10.1093/nar/gku1161 PMC4383959 25414341

[2] Attaf M Legut M Cole DK Sewell AK. The T Cell Antigen Receptor: The Swiss Army Knife of the Immune System. Clin Exp Immunol (2015) 181(1):1–18. doi: 10.1111/cei.12622 25753381 PMC4469151

[3] Simpson AA Mohammed F Salim M Tranter A Rickinson AB Stauss HJ. Structural and Energetic Evidence for Highly Peptide-Specific Tumor Antigen Targeting *via* Allo-MHC Restriction. Proc Natl Acad Sci USA (2011) 108(52):21176–81. doi: 10.1073/pnas.1108422109 PMC3248497 22160697

[4] Wang Y Singh NK Spear TT Hellman LM Piepenbrink KH McMahan RH. How an Alloreactive T-Cell Receptor Achieves Peptide and MHC Specificity. Proc Natl Acad Sci USA (2017) 114(24):E4792–801. doi: 10.1073/pnas.1700459114 PMC5474831 28572406

[5] DeWolf S Grinshpun B Savage T Lau SP Obradovic A Shonts B. Quantifying Size and Diversity of the Human T Cell Alloresponse. JCI Insight (2018) 3(15):e121256. doi: 10.1172/jci.insight.121256 PMC6129121 30089728

[6] Zerrahn J Held W Raulet DH. The MHC Reactivity of the T Cell Repertoire Prior to Positive and Negative Selection. Cell (1997) 88(5):627–36. doi: 10.1016/S0092-8674(00)81905-4 9054502

[7] Jameson SC Hogquist KA Bevan MJ. Positive Selection of Thymocytes. Annu Rev Immunol (1995) 13:93–126. doi: 10.1146/annurev.iy.13.040195.000521 7612239

[8] Bhardwaj V Kumar V Geysen HM Sercarz EE. Degenerate Recognition of a Dissimilar Antigenic Peptide by Myelin Basic Protein-Reactive T Cells. Implications for Thymic Education and Autoimmunity. J Immunol (Baltimore Md 1950) (1993) 151(9):5000–10. 7691962

[9] Pullen AM Kappler JW Marrack P. Tolerance to Self Antigens Shapes the T-Cell Repertoire. Immunol Rev (1989) 107:125–39. doi: 10.1111/j.1600-065X.1989.tb00006.x 2522084

[10] Pai JA Satpathy AT. High-Throughput and Single-Cell T Cell Receptor Sequencing Technologies. Nat Methods (2021) 18(8):881–92. doi: 10.1038/s41592-021-01201-8 PMC9345561 34282327

[11] Papadopoulou M Sanchez Sanchez G Vermijlen D. Innate and Adaptive γδ T Cells: How, When, and Why. Immunol Rev (2020) 298(1):99–116. doi: 10.1111/imr.12926 33146423

[12] Davis MM Boyd SD. Recent Progress in the Analysis of αβt Cell and B Cell Receptor Repertoires. Curr Opin Immunol (2019) 59:109–14. doi: 10.1016/j.coi.2019.05.012 PMC7075470 31326777

[13] Sewell AK. Why Must T Cells be Cross-Reactive? Nat Rev Immunol (2012) 12(9):669–77. doi: 10.1038/nri3279 PMC7097784 22918468

[14] Klein L Kyewski B Allen PM Hogquist KA. Positive and Negative Selection of the T Cell Repertoire: What Thymocytes See (and Don’t See). Nat Rev Immunol (2014) 14(6):377–91. doi: 10.1038/nri3667 PMC4757912 24830344

[15] Frantzeskakis M Takahama Y Ohigashi I. The Role of Proteasomes in the Thymus. Front Immunol (2021) 12:646209. doi: 10.3389/fimmu.2021.646209 33815406 PMC8017227

[16] Qi Q Liu Y Cheng Y Glanville J Zhang D Lee JY. Diversity and Clonal Selection in the Human T-Cell Repertoire. Proc Natl Acad Sci USA (2014) 111(36):13139–44. doi: 10.1073/pnas.1409155111 PMC4246948 25157137

[17] Vanhanen R Heikkilä N Aggarwal K Hamm D Tarkkila H Pätilä T. T Cell Receptor Diversity in the Human Thymus. Mol Immunol (2016) 76:116–22. doi: 10.1016/j.molimm.2016.07.002 27442982

[18] Clambey ET Davenport B Kappler JW Marrack P Homann D. Molecules in Medicine Mini Review: The αβ T Cell Receptor. J Mol Med (Berl) (2014) 92(7):735–41. doi: 10.1007/s00109-014-1145-2 PMC4269364 24848996

[19] Khosravi-Maharlooei M Obradovic A Misra A Motwani K Holzl M Seay HR. Crossreactive Public TCR Sequences Undergo Positive Selection in the Human Thymic Repertoire. J Clin Invest (2019) 129(6):2446–62. doi: 10.1172/JCI124358 PMC6546456 30920391

[20] Park JE Botting RA Domínguez Conde C Popescu DM Lavaert M Kunz DJ. A Cell Atlas of Human Thymic Development Defines T Cell Repertoire Formation. Sci (New York NY) (2020) 367(6480):eaay3224. doi: 10.1101/2020.01.28.911115 PMC7611066 32079746

[21] Mallis RJ Bai K Arthanari H Hussey RE Handley M Li Z. Pre-TCR Ligand Binding Impacts Thymocyte Development Before αβtcr Expression. Proc Natl Acad Sci USA (2015) 112(27):8373–8. doi: 10.1073/pnas.1504971112 PMC4500245 26056289

[22] Tuulasvaara A Baussand J Laine P Paulin L Salminen J Auvinen P. High-Sequence Diversity and Structural Conservation in the Human T-Cell Receptor β Junctional Region During Thymic Development. Eur J Immunol (2013) 43(8):2185–93. doi: 10.1002/eji.201343360 23670527

[23] Petrie HT Livak F Schatz DG Strasser A Crispe IN Shortman K. Multiple Rearrangements in T Cell Receptor Alpha Chain Genes Maximize the Production of Useful Thymocytes. J Exp Med (1993) 178(2):615–22. doi: 10.1084/jem.178.2.615 PMC2191132 8393478

[24] Niederberger N Holmberg K Alam SM Sakati W Naramura M Gu H. Allelic Exclusion of the TCR Alpha-Chain is an Active Process Requiring TCR-Mediated Signaling and C-Cbl. J Immunol (Baltimore Md 1950) (2003) 170(9):4557–63. doi: 10.4049/jimmunol.170.9.4557 12707333

[25] Corthay A Nandakumar KS Holmdahl R. Evaluation of the Percentage of Peripheral T Cells With Two Different T Cell Receptor Alpha-Chains and of Their Potential Role in Autoimmunity. J Autoimmun (2001) 16(4):423–9. doi: 10.1006/jaut.2001.0504 11437490

[26] Li Y Teteloshvili N Tan S Rao S Han A Yang YG. Humanized Mice Reveal New Insights Into the Thymic Selection of Human Autoreactive CD8(+) T Cells. Front Immunol (2019) 10:63. doi: 10.3389/fimmu.2019.00063 30778347 PMC6369192

[27] Hou X Zeng P Zhang X Chen J Liang Y Yang J. Shorter TCR β-Chains Are Highly Enriched During Thymic Selection and Antigen-Driven Selection. Front Immunol (2019) 10:299. doi: 10.3389/fimmu.2019.00299 30863407 PMC6399399

[28] Miron M Meng W Rosenfeld AM Dvorkin S Poon MML Lam N. Maintenance of the Human Memory T Cell Repertoire by Subset and Tissue Site. Genome Med (2021) 13(1):100. doi: 10.1186/s13073-021-00918-7 34127056 PMC8204429

[29] Li HM Hiroi T Zhang Y Shi A Chen G De S. Tcrβ Repertoire of CD4+ and CD8+ T Cells is Distinct in Richness, Distribution, and CDR3 Amino Acid Composition. J Leukoc Biol (2016) 99(3):505–13. doi: 10.1189/jlb.6A0215-071RR PMC5338248 26394815

[30] Robins HS Srivastava SK Campregher PV Turtle CJ Andriesen J Riddell SR. Overlap and Effective Size of the Human CD8+ T Cell Receptor Repertoire. Sci Transl Med (2010) 2(47):47ra64. doi: 10.1126/scitranslmed.3001442 PMC3212437 20811043

[31] Kitaura K Shini T Matsutani T Suzuki R. A New High-Throughput Sequencing Method for Determining Diversity and Similarity of T Cell Receptor (TCR) α and β Repertoires and Identifying Potential New Invariant TCR α Chains. BMC Immunol (2016) 17(1):38. doi: 10.1186/s12865-016-0177-5 27729009 PMC5059964

[32] Zvyagin IV Pogorelyy MV Ivanova ME Komech EA Shugay M Bolotin DA. Distinctive Properties of Identical Twins’ TCR Repertoires Revealed by High-Throughput Sequencing. Proc Natl Acad Sci USA (2014) 111(16):5980–5. doi: 10.1073/pnas.1319389111 PMC4000852 24711416

[33] Tanno H Gould TM McDaniel JR Cao W Tanno Y Durrett RE. Determinants Governing T Cell Receptor α/β-Chain Pairing in Repertoire Formation of Identical Twins. Proc Natl Acad Sci USA (2020) 117(1):532–40. doi: 10.1073/pnas.1915008117 PMC6955297 31879353

[34] Hawes GE Struyk L van den Elsen PJ. Differential Usage of T Cell Receptor V Gene Segments in CD4+ and CD8+ Subsets of T Lymphocytes in Monozygotic Twins. J Immunol (Baltimore Md 1950) (1993) 150(5):2033–45. 8436833

[35] Malhotra U Spielman R Concannon P. Variability in T Cell Receptor V Beta Gene Usage in Human Peripheral Blood Lymphocytes. Studies of Identical Twins, Siblings, and Insulin-Dependent Diabetes Mellitus Patients. J Immunol (Baltimore Md 1950) (1992) 149(5):1802–8. 1387153

[36] Kohsaka H Taniguchi A Chen PP Ollier WE Carson DA. The Expressed T Cell Receptor V Gene Repertoire of Rheumatoid Arthritis Monozygotic Twins: Rapid Analysis by Anchored Polymerase Chain Reaction and Enzyme-Linked Immunosorbent Assay. Eur J Immunol (1993) 23(8):1895–901. doi: 10.1002/eji.1830230825 8344352

[37] Reinhardt C Melms A. Skewed TCRV Beta Repertoire in Human Thymus Persists After Thymic Emigration: Influence of Genomic Imposition, Thymic Maturation and Environmental Challenge on Human TCRV Beta Usage. Vivo Immunobiol (1998) 199(1):74–86. doi: 10.1016/S0171-2985(98)80065-X 9717669

[38] Heikkilä N Vanhanen R Yohannes DA Kleino I Mattila IP Saramäki J. Human Thymic T Cell Repertoire is Imprinted With Strong Convergence to Shared Sequences. Mol Immunol (2020) 127:112–23. doi: 10.1016/j.molimm.2020.09.003 32961421

[39] Sharon E Sibener LV Battle A Fraser HB Garcia KC Pritchard JK. Genetic Variation in MHC Proteins is Associated With T Cell Receptor Expression Biases. Nat Genet (2016) 48(9):995–1002. doi: 10.1038/ng.3625 27479906 PMC5010864

[40] Rubelt F Bolen CR McGuire HM Vander Heiden JA Gadala-Maria D Levin M. Individual Heritable Differences Result in Unique Cell Lymphocyte Receptor Repertoires of Naïve and Antigen-Experienced Cells. Nat Commun (2016) 7:11112. doi: 10.1038/ncomms11112 27005435 PMC5191574

[41] Heikkilä N Vanhanen R Yohannes DA Saavalainen P Meri S Jokiranta TS. Identifying the Inheritable Component of Human Thymic T Cell Repertoire Generation in Monozygous Twins. Eur J Immunol (2020) 50(5):748–51. doi: 10.1002/eji.201948404 31872865

[42] DeWitt WS Smith A Schoch G Hansen JA Matsen FAT Bradley P. Human T Cell Receptor Occurrence Patterns Encode Immune History, Genetic Background, and Receptor Specificity. Elife (2018) 7:e38358. doi: 10.7554/eLife.38358 30152754 PMC6162092

[43] Li H Ye C Ji G Wu X Xiang Z Li Y. Recombinatorial Biases and Convergent Recombination Determine Interindividual Tcrβ Sharing in Murine Thymocytes. J Immunol (Baltimore Md 1950) (2012) 189(5):2404–13. doi: 10.4049/jimmunol.1102087 22826324

[44] Furmanski AL Ferreira C Bartok I Dimakou S Rice J Stevenson FK. Public T Cell Receptor Beta-Chains are Not Advantaged During Positive Selection. J Immunol (Baltimore Md 1950) (2008) 180(2):1029–39. doi: 10.4049/jimmunol.180.2.1029 18178843

[45] Madi A Shifrut E Reich-Zeliger S Gal H Best K Ndifon W. T-Cell Receptor Repertoires Share a Restricted Set of Public and Abundant CDR3 Sequences That are Associated With Self-Related Immunity. Genome Res (2014) 24(10):1603–12. doi: 10.1101/gr.170753.113 PMC4199372 25024161

[46] Matsutani T Ogata M Fujii Y Kitaura K Nishimoto N Suzuki R. Shortening of Complementarity Determining Region 3 of the T Cell Receptor α Chain During Thymocyte Development. Mol Immunol (2011) 48(4):623–9. doi: 10.1016/j.molimm.2010.11.003 21126768

[47] Wang C Sanders CM Yang Q Schroeder HW Jr. Wang E Babrzadeh F. High Throughput Sequencing Reveals a Complex Pattern of Dynamic Interrelationships Among Human T Cell Subsets. Proc Natl Acad Sci USA (2010) 107(4):1518–23. doi: 10.1073/pnas.0913939107 PMC2824416 20080641

[48] Dahal-Koirala S Risnes LF Neumann RS Christophersen A Lundin KEA Sandve GK. Comprehensive Analysis of CDR3 Sequences in Gluten-Specific T-Cell Receptors Reveals a Dominant R-Motif and Several New Minor Motifs. Front Immunol (2021) 12:639672. doi: 10.3389/fimmu.2021.639672 33927715 PMC8076556

[49] Heikkilä N Sormunen S Mattila J Härkönen T Knip M Ihantola EL. Generation of Self-Reactive, Shared T-Cell Receptor α Chains in the Human Thymus. J Autoimmun (2021) 119:102616. doi: 10.1016/j.jaut.2021.102616 33652347

[50] Zhao Y Nguyen P Ma J Wu T Jones LL Pei D. Preferential Use of Public TCR During Autoimmune Encephalomyelitis. J Immunol (Baltimore Md 1950) (2016) 196(12):4905–14. doi: 10.4049/jimmunol.1501029 PMC4902001 27183575

[51] Gomez-Tourino I Kamra Y Baptista R Lorenc A Peakman M. T Cell Receptor β-Chains Display Abnormal Shortening and Repertoire Sharing in Type 1 Diabetes. Nat Commun (2017) 8(1):1792. doi: 10.1038/s41467-017-01925-2 29176645 PMC5702608

[52] Reiser JB Darnault C Grégoire C Mosser T Mazza G Kearney A. CDR3 Loop Flexibility Contributes to the Degeneracy of TCR Recognition. Nat Immunol (2003) 4(3):241–7. doi: 10.1038/ni891 12563259

[53] Wooldridge L Ekeruche-Makinde J van den Berg HA Skowera A Miles JJ Tan MP. A Single Autoimmune T Cell Receptor Recognizes More Than a Million Different Peptides. J Biol Chem (2012) 287(2):1168–77. doi: 10.1074/jbc.M111.289488 PMC3256900 22102287

[54] Koncz B Balogh GM Papp BT Asztalos L Kemény L Manczinger M. Self-Mediated Positive Selection of T Cells Sets an Obstacle to the Recognition of Nonself. Proc Natl Acad Sci USA (2021) 118(37):e2100542118. doi: 10.1073/pnas.2100542118 34507984 PMC8449404

[55] Carter JA Preall JB Grigaityte K Goldfless SJ Jeffery E Briggs AW. Single T Cell Sequencing Demonstrates the Functional Role of αβ TCR Pairing in Cell Lineage and Antigen Specificity. Front Immunol (2019) 10:1516. doi: 10.3389/fimmu.2019.01516 31417541 PMC6684766

[56] Pham HP Manuel M Petit N Klatzmann D Cohen-Kaminsky S Six A. Half of the T-Cell Repertoire Combinatorial Diversity is Genetically Determined in Humans and Humanized Mice. Eur J Immunol (2012) 42(3):760–70. doi: 10.1002/eji.201141798 22105329

[57] Marodon G Desjardins D Mercey L Baillou C Parent P Manuel M. High Diversity of the Immune Repertoire in Humanized NOD.SCID.gamma C-/- Mice. Eur J Immunol (2009) 39(8):2136–45. doi: 10.1002/eji.200939480 19572320

[58] Vandekerckhove BA Baccala R Jones D Kono DH Theofilopoulos AN Roncarolo MG. Thymic Selection of the Human T Cell Receptor V Beta Repertoire in SCID-Hu Mice. J Exp Med (1992) 176(6):1619–24. doi: 10.1084/jem.176.6.1619 PMC2119440 1460421

[59] Shimizu I Fudaba Y Shimizu A Yang YG Sykes M. Comparison of Human T Cell Repertoire Generated in Xenogeneic Porcine and Human Thymus Grafts. Transplantation (2008) 86(4):601–10. doi: 10.1097/TP.0b013e318182d47a PMC2680689 18724231

[60] Khosravi-Maharlooei M Hoelzl M Li HW Madley RC Waffarn EE Danzl NM. Rapid Thymectomy of NSG Mice to Analyze the Role of Native and Grafted Thymi in Humanized Mice. Eur J Immunol (2020) 50(1):138–41. doi: 10.1002/eji.201948205 PMC6940512 31583677

[61] Redondo MJ Yu L Hawa M Mackenzie T Pyke DA Eisenbarth GS. Heterogeneity of Type I Diabetes: Analysis of Monozygotic Twins in Great Britain and the United States. Diabetologia (2001) 44(3):354–62. doi: 10.1007/s001250051626 11317668

[62] Stadinski BD Shekhar K Gómez-Touriño I Jung J Sasaki K Sewell AK. Hydrophobic CDR3 Residues Promote the Development of Self-Reactive T Cells. Nat Immunol (2016) 17(8):946–55. doi: 10.1038/ni.3491 PMC4955740 27348411

[63] Han A Glanville J Hansmann L Davis MM. Linking T-Cell Receptor Sequence to Functional Phenotype at the Single-Cell Level. Nat Biotechnol (2014) 32(7):684–92. doi: 10.1038/nbt.2938 PMC4337815 24952902

[64] Glanville J Huang H Nau A Hatton O Wagar LE Rubelt F. Identifying Specificity Groups in the T Cell Receptor Repertoire. Nature (2017) 547(7661):94–8. doi: 10.1038/nature22976 PMC5794212 28636589

[65] Huang H Wang C Rubelt F Scriba TJ Davis MM. Analyzing the Mycobacterium Tuberculosis Immune Response by T-Cell Receptor Clustering With GLIPH2 and Genome-Wide Antigen Screening. Nat Biotechnol (2020) 38(10):1194–202. doi: 10.1038/s41587-020-0505-4 PMC7541396 32341563

[66] DeWolf S Sykes M. Alloimmune T Cells in Transplantation. J Clin Invest (2017) 127(7):2473–81. doi: 10.1172/JCI90595 PMC5490749 28628037

[67] Sha WC Nelson CA Newberry RD Kranz DM Russell JH Loh DY. Positive and Negative Selection of an Antigen Receptor on T Cells in Transgenic Mice. Nature (1988) 336(6194):73–6. doi: 10.1038/336073a0 3263574

[68] Manilay JO Pearson DA Sergio JJ Swenson KG Sykes M. Intrathymic Deletion of Alloreactive T Cells in Mixed Bone Marrow Chimeras Prepared With a Nonmyeloablative Conditioning Regimen. Transplantation (1998) 66(1):96–102. doi: 10.1097/00007890-199807150-00015 9679828

[69] Tomita Y Khan A Sykes M. Role of Intrathymic Clonal Deletion and Peripheral Anergy in Transplantation Tolerance Induced by Bone Marrow Transplantation in Mice Conditioned With a Nonmyeloablative Regimen. J Immunol (Baltimore Md 1950) (1994) 153(3):1087–98. 8027542

[70] Altman JD Moss PA Goulder PJ Barouch DH McHeyzer-Williams MG Bell JI. Phenotypic Analysis of Antigen-Specific T Lymphocytes. Sci (New York NY) (1996) 274(5284):94–6. doi: 10.1126/science.274.5284.94 8810254

[71] Mutis T Gillespie G Schrama E Falkenburg JH Moss P Goulmy E. Tetrameric HLA Class I-Minor Histocompatibility Antigen Peptide Complexes Demonstrate Minor Histocompatibility Antigen-Specific Cytotoxic T Lymphocytes in Patients With Graft-Versus-Host Disease. Nat Med (1999) 5(7):839–42. doi: 10.1038/10563 10395333

[72] Bach F Hirschhorn K. LYMPHOCYTE INTERACTION: A POTENTIAL HISTOCOMPATIBILITY TEST IN VITRO. Sci (New York NY) (1964) 143(3608):813–4. doi: 10.1126/science.143.3608.813 14088078

[73] Rubin AL Stenzel KH Hirschhorn K Bach F. HISTOCOMPATIBILITY AND IMMUNOLOGIC COMPETENCE IN RENAL HOMOTRANSPLANTATION. Sci (New York NY) (1964) 143(3608):815–6. doi: 10.1126/science.143.3608.815 14088079

[74] Goulmy E Stijnen T Groenewoud AF Persijn GG Blokland E Pool J. Renal Transplant Patients Monitored by the Cell-Mediated Lympholysis Assay. Evaluation of its Clinical Value. Transplantation (1989) 48(4):559–63. doi: 10.1097/00007890-198910000-00004 2799908

[75] Schwarer AP Jiang YZ Brookes PA Barrett AJ Batchelor JR Goldman JM. Frequency of Anti-Recipient Alloreactive Helper T-Cell Precursors in Donor Blood and Graft-Versus-Host Disease After HLA-Identical Sibling Bone-Marrow Transplantation. Lancet (1993) 341(8839):203–5. doi: 10.1016/0140-6736(93)90067-Q 8093498

[76] Heeger PS Greenspan NS Kuhlenschmidt S Dejelo C Hricik DE Schulak JA. Pretransplant Frequency of Donor-Specific, IFN-Gamma-Producing Lymphocytes is a Manifestation of Immunologic Memory and Correlates With the Risk of Posttransplant Rejection Episodes. J Immunol (Baltimore Md 1950) (1999) 163(4):2267–75. 10438971

[77] Ashoor I Najafian N Korin Y Reed EF Mohanakumar T Ikle D. Standardization and Cross Validation of Alloreactive IFNgamma ELISPOT Assays Within the Clinical Trials in Organ Transplantation Consortium. Am J Transplant Off J Am Soc Transplant Am Soc Transplant Surgeons (2013) 13(7):1871–9. doi: 10.1111/ajt.12286 PMC3839289 23710568

[78] Streilein JW Strome P Wood PJ. Failure of *In Vitro* Assays to Predict Accurately the Existence of Neonatally Induced H-2 Tolerance. Transplantation (1989) 48(4):630–4. 2529680

[79] Najafian N Albin MJ Newell KA. How can We Measure Immunologic Tolerance in Humans? J Am Soc Nephrol (2006) 17(10):2652–63. doi: 10.1681/ASN.2005070707 16928808

[80] Morris H DeWolf S Robins H Sprangers B LoCascio SA Shonts BA. Tracking Donor-Reactive T Cells: Evidence for Clonal Deletion in Tolerant Kidney Transplant Patients. Sci Transl Med (2015) 7(272):272ra10. doi: 10.1126/scitranslmed.3010760 PMC4360892 25632034

[81] Currier JR Robinson MA. Spectratype/immunoscope Analysis of the Expressed TCR Repertoire. Curr Protoc Immunol (2001) Chapter 10:Unit 10.28. doi: 10.1002/0471142735.im1028s38 18432693

[82] Ciupe SM Devlin BH Markert ML Kepler TB. Quantification of Total T-Cell Receptor Diversity by Flow Cytometry and Spectratyping. BMC Immunol (2013) 14:35. doi: 10.1186/1471-2172-14-35 23914737 PMC3750526

[83] Kitaura K Fujii Y Matsutani T Shirai K Suzuki S Takasaki T. A New Method for Quantitative Analysis of the T Cell Receptor V Region Repertoires in Healthy Common Marmosets by Microplate Hybridization Assay. J Immunol Methods (2012) 384(1-2):81–91. doi: 10.1016/j.jim.2012.07.012 22841578

[84] Robins HS Campregher PV Srivastava SK Wacher A Turtle CJ Kahsai O. Comprehensive Assessment of T-Cell Receptor Beta-Chain Diversity in Alphabeta T Cells. Blood (2009) 114(19):4099–107. doi: 10.1182/blood-2009-04-217604 PMC2774550 19706884

[85] Freeman JD Warren RL Webb JR Nelson BH Holt RA. Profiling the T-Cell Receptor Beta-Chain Repertoire by Massively Parallel Sequencing. Genome Res (2009) 19(10):1817–24. doi: 10.1101/gr.092924.109 PMC2765271 19541912

[86] Newell EW Davis MM. Beyond Model Antigens: High-Dimensional Methods for the Analysis of Antigen-Specific T Cells. Nat Biotechnol (2014) 32(2):149–57. doi: 10.1038/nbt.2783 PMC4001742 24441473

[87] Macedo C Orkis EA Popescu I Elinoff BD Zeevi A Shapiro R. Contribution of Naive and Memory T-Cell Populations to the Human Alloimmune Response. Am J Transplant Off J Am Soc Transplant Am Soc Transplant Surgeons (2009) 9(9):2057–66. doi: 10.1111/j.1600-6143.2009.02742.x 19624567

[88] Golshayan D Wyss JC Buckland M Hernandez-Fuentes M Lechler RI. Differential Role of Naive and Memory CD4 T-Cell Subsets in Primary Alloresponses. Am J Transplant Off J Am Soc Transplant Am Soc Transplant Surgeons (2010) 10(8):1749–59. doi: 10.1111/j.1600-6143.2010.03180.x 20659087

[89] Oakes T Heather JM Best K Byng-Maddick R Husovsky C Ismail M. Quantitative Characterization of the T Cell Receptor Repertoire of Naïve and Memory Subsets Using an Integrated Experimental and Computational Pipeline Which Is Robust, Economical, and Versatile. Front Immunol (1267) 2017:8. doi: 10.3389/fimmu.2017.01267 PMC5643411 29075258

[90] Melenhorst JJ Scheinberg P Williams A Ambrozak DR Keyvanfar K Smith M. Alloreactivity Across HLA Barriers Is Mediated by Both Naïve and Antigen-Experienced T Cells. Biol Blood Marrow Transplantation (2011) 17(6):800–9. doi: 10.1016/j.bbmt.2010.12.711 PMC3100442 21215812

[91] Obradovic A Shen Y Sykes M Fu J. Integrated Analysis Toolset for Defining and Tracking Alloreactive T-Cell Clones After Human Solid Organ and Hematopoietic Stem Cell Transplantation. Software Impacts (2021) 10:100142. doi: 10.1016/j.simpa.2021.100142 PMC8920412 35291378

[92] Aschauer C Jelencsics K Hu K Heinzel A Vetter J Fraunhofer T. Next Generation Sequencing Based Assessment of the Alloreactive T Cell Receptor Repertoire in Kidney Transplant Patients During Rejection: A Prospective Cohort Study. BMC Nephrol (2019) 20(1):346. doi: 10.1186/s12882-019-1541-5 31477052 PMC6719356

[93] Savage TM Shonts BA Lau S Obradovic A Robins H Shaked A. Deletion of Donor-Reactive T Cell Clones After Human Liver Transplant. Am J Transplant Off J Am Soc Transplant Am Soc Transplant Surgeons (2020) 20(2):538–45. doi: 10.1111/ajt.15592 PMC6984984 31509321

[94] Zuber J Shonts B Lau SP Obradovic A Fu J Yang S. Bidirectional Intragraft Alloreactivity Drives the Repopulation of Human Intestinal Allografts and Correlates With Clinical Outcome. Sci Immunol (2016) 1(4):eaah3732. doi: 10.1126/sciimmunol.aah3732 28239678 PMC5323244

[95] Fu J Zuber J Martinez M Shonts B Obradovic A Wang H. Human Intestinal Allografts Contain Functional Hematopoietic Stem and Progenitor Cells That Are Maintained by a Circulating Pool. Cell Stem Cell (2019) 24(2):227–39. doi: 10.1016/j.stem.2018.11.007 PMC6398344 30503142

[96] Fu J Zuber J Shonts B Obradovic A Wang Z Frangaj K. Lymphohematopoietic Graft-Versus-Host Responses Promote Mixed Chimerism in Patients Receiving Intestinal Transplantation. J Clin Invest (2021) 131(8):e141698. doi: 10.1172/JCI141698 PMC8062082 33630757

[97] Aschauer C Jelencsics K Hu K Heinzel A Gregorich MG Vetter J. Prospective Tracking of Donor-Reactive T-Cell Clones in the Circulation and Rejecting Human Kidney Allografts. Front Immunol (2021) 12(4153). doi: 10.3389/fimmu.2021.750005 PMC8552542 34721420

[98] Smith JM Weaver T Skeans MA Horslen SP Harper AM Snyder JJ. OPTN/SRTR 2016 Annual Data Report: Intestine. Am J Transplant Off J Am Soc Transplant Am Soc Transplant Surgeons (2018) 18 Suppl 1:254–90. doi: 10.1111/ajt.14560 29292606

[99] Fishbein TM. Intestinal Transplantation. New Engl J Med (2009) 361(10):998–1008. doi: 10.1056/NEJMra0804605 19726774

[100] Sudan D. The Current State of Intestine Transplantation: Indications, Techniques, Outcomes and Challenges. Am J Transplant Off J Am Soc Transplant Am Soc Transplant Surgeons (2014) 14(9):1976–84. doi: 10.1111/ajt.12812 25307033

[101] Trentadue G Dijkstra G. Current Understanding of Alloimmunity of the Intestinal Graft. Curr Opin Organ Transplant (2015) 20(3):286–94. doi: 10.1097/MOT.0000000000000196 25944233

[102] Kato T Tzakis AG Selvaggi G Gaynor JJ David AI Bussotti A. Intestinal and Multivisceral Transplantation in Children. Ann Surg (2006) 243(6):756–64; discussion 64-6. doi: 10.1097/01.sla.0000219696.11261.13 16772779 PMC1570576

[103] Abu-Elmagd KM Kosmach-Park B Costa G Zenati M Martin L Koritsky DA. Long-Term Survival, Nutritional Autonomy, and Quality of Life After Intestinal and Multivisceral Transplantation. Ann Surg (2012) 256(3):494–508. doi: 10.1097/SLA.0b013e318265f310 22868368

[104] Zuber J Rosen S Shonts B Sprangers B Savage TM Richman S. Macrochimerism in Intestinal Transplantation: Association With Lower Rejection Rates and Multivisceral Transplants, Without GVHD. Am J Transplant Off J Am Soc Transplant Am Soc Transplant Surgeons (2015) 15(10):2691–703. doi: 10.1111/ajt.13325 PMC4575629 25988811

[105] Pelot MR Pearson DA Swenson K Zhao G Sachs J Yang YG. Lymphohematopoietic Graft-vs.-Host Reactions can be Induced Without Graft-vs.-Host Disease in Murine Mixed Chimeras Established With a Cyclophosphamide-Based Nonmyeloablative Conditioning Regimen. Biol Blood marrow Transplant J Am Soc Blood Marrow Transplantation (1999) 5(3):133–43. doi: 10.1053/bbmt.1999.v5.pm10392959 10392959

[106] Sykes M Sheard MA Sachs DH. Graft-Versus-Host-Related Immunosuppression is Induced in Mixed Chimeras by Alloresponses Against Either Host or Donor Lymphohematopoietic Cells. J Exp Med (1988) 168(6):2391–6. doi: 10.1084/jem.168.6.2391 PMC2189158 3264329

[107] Fu J Wang Z Martinez M Frangaj K Jones R Guo X. O-40: Single-Cell Immune Profiling of Human Intestinal Allografts Reveals Differential Contributions of HvG T-Cell Clones in Quiescent vs Chronically Rejecting Allografts. Transplantation (2021) 105(7S):S21. doi: 10.1097/01.tp.0000757628.93991.24 34792971

[108] Stuart T Butler A Hoffman P Hafemeister C Papalexi E Mauck WM 3rd. Comprehensive Integration of Single-Cell Data. Cell (2019) 177(7):1888–902.e21. doi: 10.1016/j.cell.2019.05.031 31178118 PMC6687398

[109] Kalyan S Kabelitz D. Defining the Nature of Human Gammadelta T Cells: A Biographical Sketch of the Highly Empathetic. Cell Mol Immunol (2013) 10(1):21–9. doi: 10.1038/cmi.2012.44 PMC4003173 23085947

[110] Khairallah C Chu TH Sheridan BS. Tissue Adaptations of Memory and Tissue-Resident Gamma Delta T Cells. Front Immunol (2018) 9:2636. doi: 10.3389/fimmu.2018.02636 30538697 PMC6277633

[111] Sant S Jenkins MR Dash P Watson KA Wang Z Pizzolla A. Human Gammadelta T-Cell Receptor Repertoire is Shaped by Influenza Viruses, Age and Tissue Compartmentalisation. Clin Transl Immunol (2019) 8(9):e1079. doi: 10.1002/cti2.1079 PMC6756999 31559018

[112] Xu W Monaco G Wong EH Tan WLW Kared H Simoni Y. Mapping of Gamma/Delta T Cells Reveals Vdelta2+ T Cells Resistance to Senescence. EBioMedicine (2019) 39:44–58. doi: 10.1016/j.ebiom.2018.11.053 30528453 PMC6354624

[113] Sullivan LC Shaw EM Stankovic S Snell GI Brooks AG Westall GP. The Complex Existence of γδ T Cells Following Transplantation: The Good, the Bad and the Simply Confusing. Clin Trans Immunol (2019) 8(9):e1078. doi: 10.1002/cti2.1078 PMC6748302 31548887

[114] Nagai M Azuma E Qi J Kumamoto T Hiratake S Hirayama M. Suppression of Alloreactivity With Gamma Delta T-Cells: Relevance to Increased Gamma Delta T-Cells Following Bone Marrow Transplantation. BioMed Pharmacother (1998) 52(3):137–42. doi: 10.1016/S0753-3322(98)80092-9 9755807

[115] Petrasca A Doherty DG. Human Vδ2(+) γδ T Cells Differentially Induce Maturation, Cytokine Production, and Alloreactive T Cell Stimulation by Dendritic Cells and B Cells. Front Immunol (2014) 5:650. doi: 10.3389/fimmu.2014.00650 25566261 PMC4271703

[116] Hunter S Willcox CR Davey MS Kasatskaya SA Jeffery HC Chudakov DM. Human Liver Infiltrating γδ T Cells are Composed of Clonally Expanded Circulating and Tissue-Resident Populations. J Hepatol (2018) 69(3):654–65. doi: 10.1016/j.jhep.2018.05.007 PMC6089840 29758330

[117] Fu J Fang Z Gorur A Jiao W Wang Z Zuber J. O-37: Immune Profiling of γδ T Cells Locally and Systemically After Human Intestinal Transplantation Reveals Unique Innate and Adaptive Features. Transplantation (2021) 105(7S):S19. doi: 10.1097/01.tp.0000757616.20365.29

